# A History of Cholecystectomy

**DOI:** 10.1097/AS9.0000000000000699

**Published:** 2026-08-20

**Authors:** Imran J. Anwar, Theodore N. Pappas

**Affiliations:** From the * Department of Surgery, Duke University, Durham, NC.

**Keywords:** cholecystectomy, cholecystostomy, cholecystitis, cholelithiasis, choledocholithiasis, history

## Abstract

Benign disease of the gallbladder is common and frequently treated by surgical removal of the gallbladder. Today, cholecystectomy is the most common operation performed by general surgeons, and the majority are done via minimally invasive techniques. However, during the early history of the surgical management of gallbladder disease, cholecystectomy was seldom performed due to perceived risks. We present a historical review of the management of benign gallbladder disease, delineating 4 distinct phases of the surgical approach to the disease. The management of benign gallbladder disease has drastically changed over the last 50 years, and innovations in the care of gallbladder patients have influenced nearly all aspects of abdominal surgery. We argue that the introduction of laparoscopic cholecystectomy was a watershed moment in the history of surgery, serving as a gateway procedure for minimally invasive surgery innovations that have occurred since.

## INTRODUCTION

Benign disease of the gallbladder is common and frequently treated by surgical removal of the gallbladder. Today, cholecystectomy is the most common operation performed by general surgeons, and the majority are done via minimally invasive techniques. However, historically, gallbladder disease was seldom managed with cholecystectomy. The history of gallbladder surgery is complicated and dates from the mid-19^th^ century to the evolving field today. The surgical approach has changed over the years, and innovations in the care of gallbladder patients have influenced nearly all abdominal surgery. There are 4 distinct phases in the history of the surgical care of benign gallbladder disease. This paper will review the 4 historical phases of the evolution of gallbladder surgery and highlight laparoscopic cholecystectomy as a “gateway procedure” that influenced the greater field of abdominal surgery in its wake.

### Phase 1. The Early Management of Benign Gallbladder Disease

The recognition that gallstones were pathologic and could lead to complications was made in the 1500s and is ascribed to a variety of investigators.^1,2^ Diagnosis at that time was limited to symptomatology, as well as physical examination, such as jaundice, fevers, and the presence of a tender right upper quadrant mass.^3^ Several generations of surgeons followed until Jean-Louis Petit (1674–1760) proposed that stones of the gallbladder might be treated in a similar fashion to stones in the urinary bladder by a procedure called lithotomy.^3–5^ In 1743, he accidentally carried out such an operation after attempting to drain what was thought to be an abdominal wall abscess.^6^ Until the 1800s, most surgeons operated on pathologies that could be dealt with externally (eg, amputation or skin tumor removal). Surgeries of the abdominal cavity were reserved for life-threatening disease, palliation, or traumatic wounds.^7^ Ephraim McDowell performed the first successful planned ovariotomy, the removal of a diseased ovary, in 1809 and ushered in abdominal surgeries for chronic rather than life-threatening conditions. With the advent of anesthesia and Lister antisepsis, favorable outcomes following ovariotomy showed that the peritoneal cavity could be safely opened.^8^

The first cholecystotomy occurred in 1867 when John Stough Bobbs (1809–1870) reported on the care of a patient with abdominal pain and a palpable mass.^9^ Bobbs thought he was operating on an ovarian mass but encountered a dilated gallbladder. He opened the dome of the gallbladder, removed fluid and stones, and closed the gallbladder. The patient recovered with resolution of symptoms.^9^ Over the next several decades, the diagnosis of gallbladder disease usually relied on a markedly distended gallbladder in a thin patient with a palpable mass. A tender mass in the right upper quadrant was either a tumor, an abscess (liver or otherwise), or a complication of cholelithiasis.^10^ At the time of Bobbs’ initial case report, most surgeons believed that the gallbladder was important to gastrointestinal function, and operations were designed to keep a functioning gallbladder.^11^ Therefore, if a patient was operated on for symptomatic gallstones, a cholecystotomy was needed to remove the stones. It was assumed that after the gallstones were removed, if there was free flow of bile, then the cystic duct was open, and therefore the gallbladder could simply be closed.^12^ If there was no free flow of bile, the surgeons searched inside of the gallbladder for the offending cystic duct stone in an effort to relieve the obstruction and keep the organ as a functioning storage site for bile.^13^ If common duct stones were palpated during exploration and the patient was jaundiced, forceps were used to crush the stone through the bile duct wall. The fragments were milked back into the gallbladder or pushed into the duodenum.^14^ Alternatively, a palpable stone in the common duct could be removed by making a hole in the common duct directly over the palpable stone, removing the stone, and closing the wall of the common duct. This method of common duct exploration was associated with a 25% postoperative mortality.^15^

It was later learned that if the cystic duct was closed, the gallbladder was best managed by simply suturing it to the skin to allow drainage of the obstructed organ, creating a cholecysto-cutaneous fistula. The patients who survived cholecystotomy and suturing of the gallbladder to the wound had 1 of 3 outcomes: if the cystic duct was open, the patient had bile drainage at the skin until the cholecysto-cutaneous fistula closed. If the fistula did not close, the result was chronic bile drainage at the skin. Alternatively, if the cystic duct was occluded, the patient often had chronic mucous drainage from the gallbladder to the skin.

Carl Langenbuch (1846–1901) was the first to report a successful cholecystectomy for the treatment of symptomatic gallstones. In July of 1882, Langenbuch explored the abdomen of a 43-year-old male with chronic biliary colic. He used a horizontal incision along the lower border of the liver joined by a vertical incision along the rectus. He initially drained the gallbladder. He then ligated the cystic duct and removed the gallbladder in its entirety. The patient recovered and left the hospital after a 6-week stay.^16^ Langenbuch published report began with a case history of another patient who did not receive an operation but died a premature death due to presumed complications of gallstone disease. Before he presented the history of the first successful cholecystectomy, he built his case for proceeding with “radical” extirpation of the gallbladder by reviewing the history of cholecystostomy, cholelithotomy, and other remedial operations for gallstone disease. He stated that these operations “treat only the end result of the disease and do not eliminate the underlying disease itself”. He further suggested that removal of the gallbladder would of little consequence given the existence of several animal species without gallbladders and prior reports of congenital absence of a gallbladder in healthy humans.

In the same publication, Langenbuch explained how he prepared to perform the first cholecystectomy by practicing on cadavers until he perfected the technique. The actual description of the operation was relatively anticlimactic in the manuscript. The operation occurred on July 15, 1882, at the Lazarus Hospital in Berlin, assisted by 2 colleagues. The patient had an uneventful postoperative course and was seen in follow-up 4 months later and was symptom-free. The second phase of the history of the surgical management of gallbladder disease began with the report of Langenbuch successful operation.^16^

### Phase 2. Open Cholecystectomy vs Cholecystotomy

**FIGURE 1.**
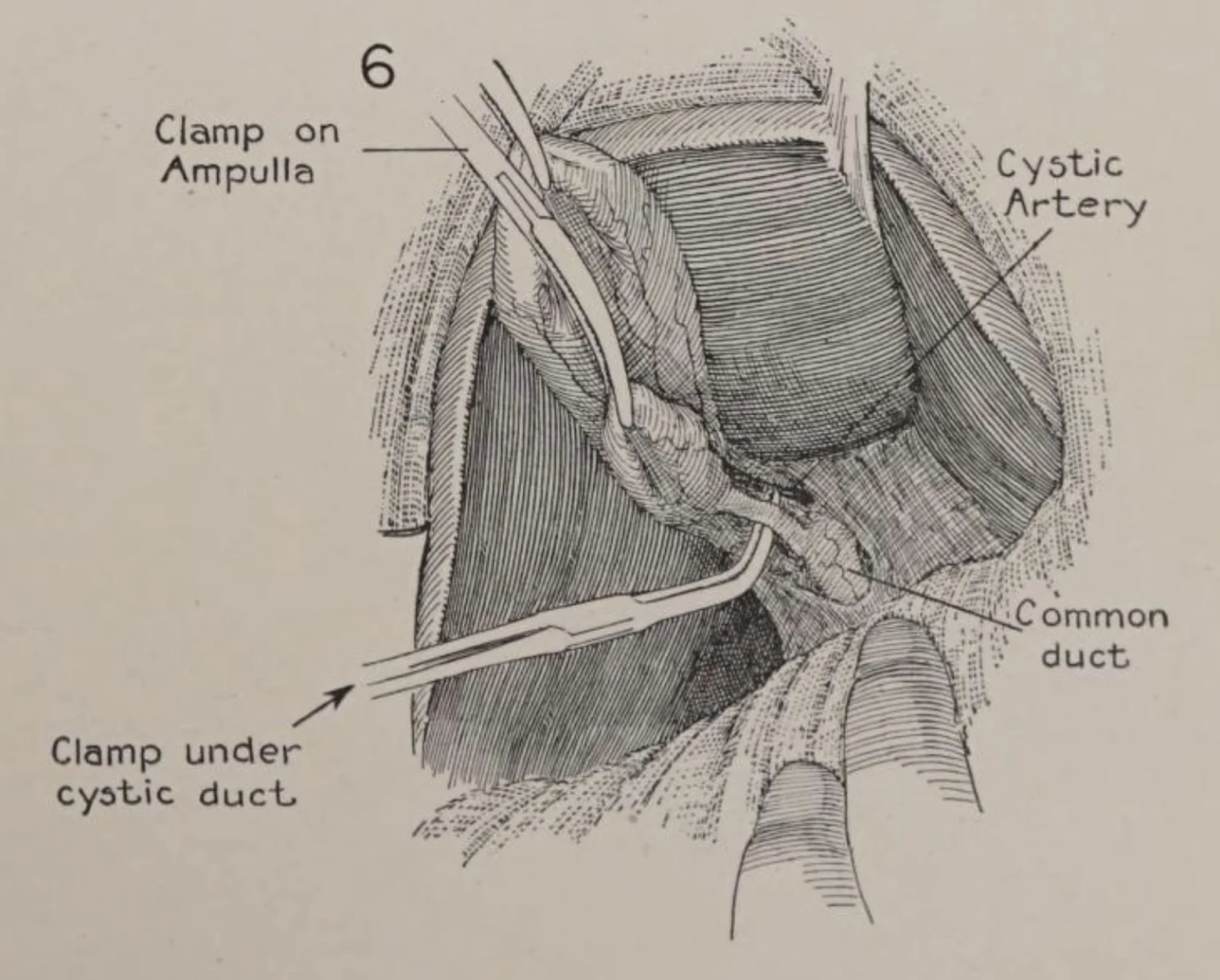
Exposure of the cystic duct and artery in an open cholecystectomy (from^17^). The infundibulum of the gallbladder is denoted as the ampulla in the figure.

**FIGURE 2.**
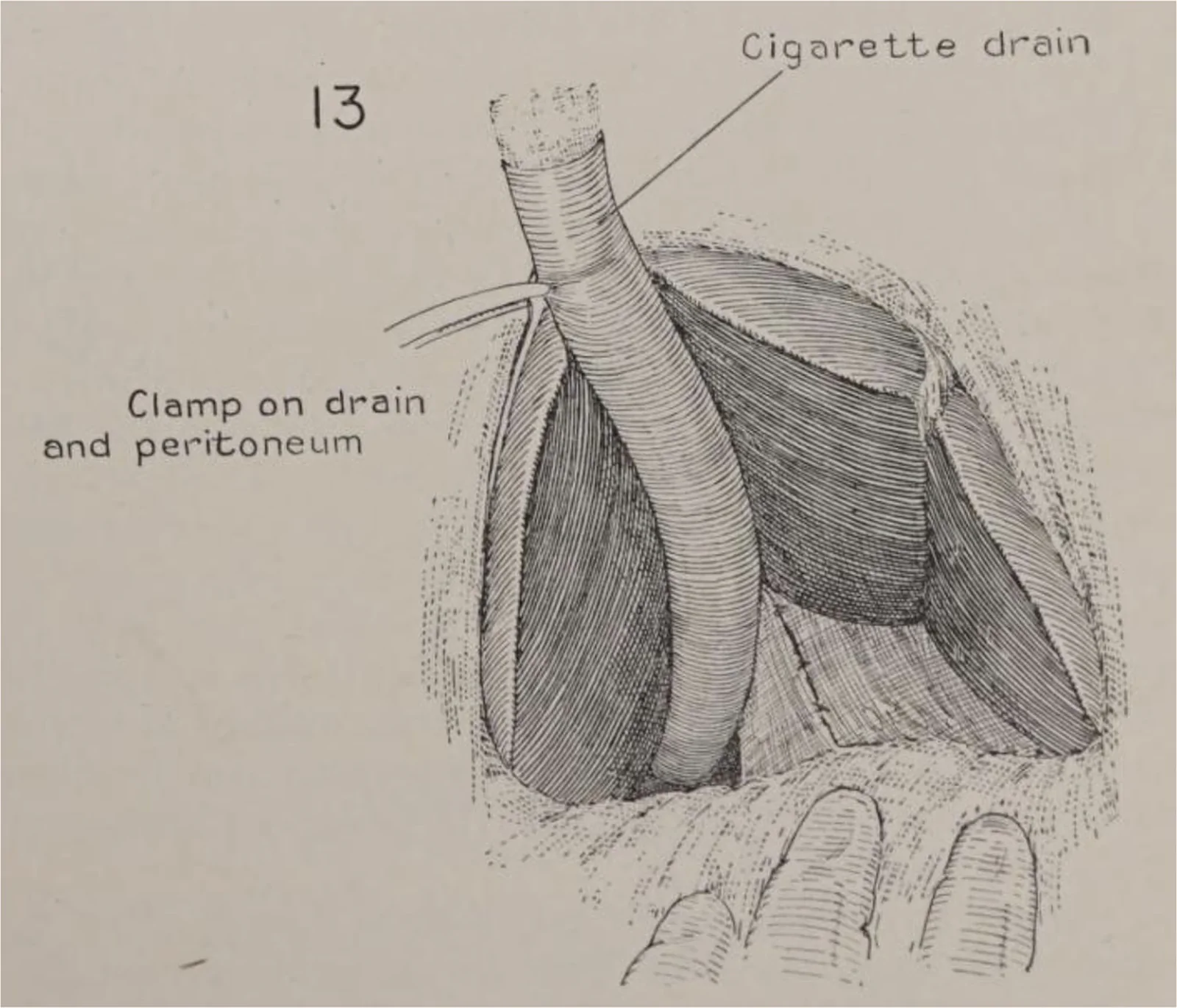
Closure of the gallbladder fossa and placement of a cigarette drain following open cholecystectomy (from^17^). A cigarette drain is a passive drain composed of a small strip of gauze placed into a rubber tube.^18^

**FIGURE 3.**
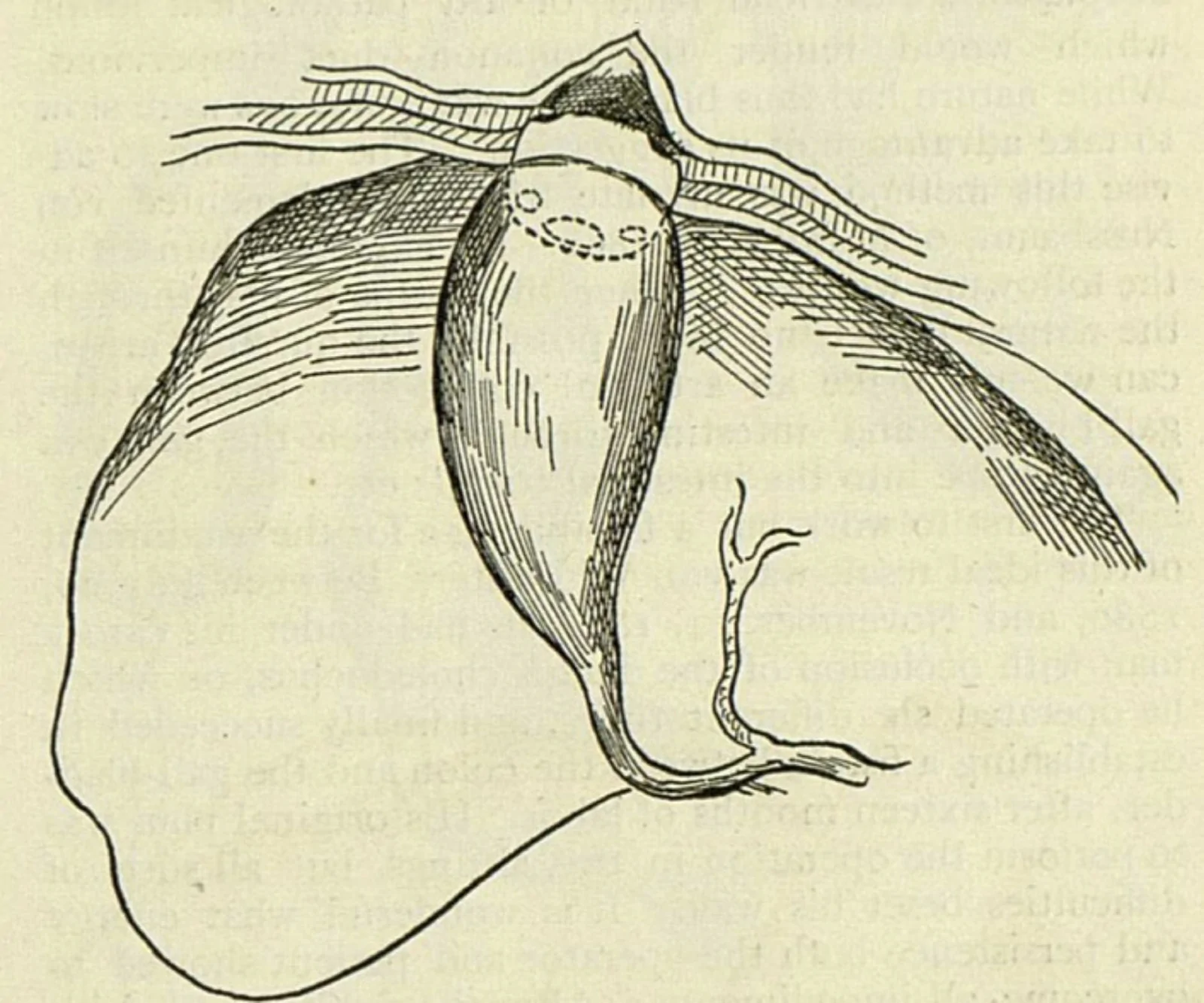
Creation of cholecystostomy (from^19^).

For 20 years after Langenbuch description of cholecystectomy, a debate raged in the surgical literature. Initially, cholecystostomy was still considered the surgical standard of care since cholecystectomy was thought to be a radical, technically challenging operation only indicated for contracted, friable gallbladders that could not be sewn to the wound.^15^ Simply sewing the dome of a distended gallbladder to the abdominal wound (ie, cholecystostomy) was not a complicated operation and was possible for surgeons of varying abilities.^11^ With time, as the technique of cholecystectomy became widely adopted, the morbidity and mortality of the operation improved. As surgeons became comfortable with both cholecystostomy and cholecystectomy, they had at least two options in the surgical care of gallbladder patients. If the gallbladder was assessed to be non-functional (eg, contracted or with cystic duct occlusion) or nonsalvageable (eg, necrotic wall), a cholecystectomy could be performed (Figs. 1 and 2). Conversely, if the cystic duct was patent and the stones could be removed, then it made sense to sew it to the skin or close the dome of the gallbladder, avoiding the possible morbidity and mortality associated with cholecystectomy (Fig. 3). Following cholecystostomy, if the stones were removed and the cystic duct was patent, the skin fistula might close, and the gallbladder would regain normal function. Most of the time, stones would not form again.

Between the 1890 and 1920, despite the availability of both cholecystectomy and cholecystostomy, most surgeons would do the simplest operation (ie, cholecystostomy) and only take out the gallbladder if absolutely necessary.^20^ The written literature was penned usually by skilled academic surgeons, and the “standard of care” only started to shift early in the 20^th^ century. Concurrently, major abdominal surgeries became increasingly possible due to the progress in anesthesia, antiseptics, and surgical techniques, allowing surgeons increasingly to experiment with procedures in the peritoneal cavity.^21^ The concept of surgical disease became broadly accepted, in part driven by the widespread adoption of appendectomy for acute appendicitis.^22^ Patients were more willing to undergo a surgical procedure, and surgeries were more likely to be offered by medical practitioners. By the 1920s, most surgeons agreed that cholecystectomy was indicated for most if not all complications of gallstone disease.^23^

**FIGURE 4.**
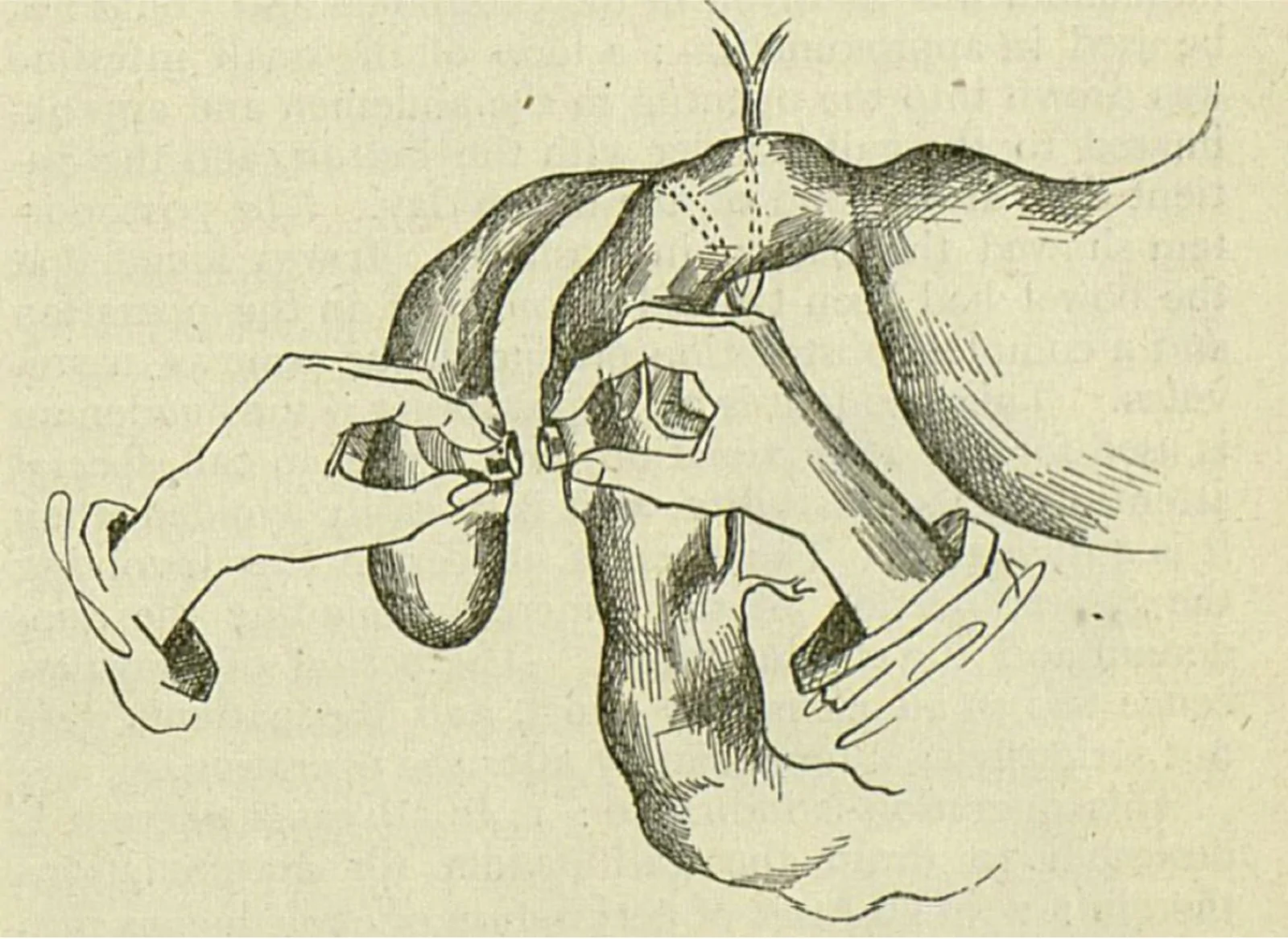
The use of Murphy button to create a cholecystoduodenostomy (from^19^).

Cholecystostomy was still indicated in select cases, such as in septic patients with severe cholecystitis. Simple drainage of the gallbladder stabilized the patient by providing source control for systemic infection, allowing for a completion cholecystectomy in the future as necessary. Other surgical approaches were still considered such as cholecysto-enterostomy (Fig. 4) but were recommended for only unique cases.^19,24^

Finally, the diagnosis of benign biliary pathologies improved in the 1920s due to progress in imaging. Evarts Graham and Warren Cole published in 1924 the first report of a cholecystography,^25^ the X-ray imaging of the gallbladder by using iodinated contrast.^25,26^ Prior to this, diagnosis of biliary disease solely relied on symptoms, physical examination, and exploratory surgery. Cholecystography allowed for diagnosis of acute cholecystitis (diagnosed by the non-opacification of the gallbladder on cholecystography) as well as cholelithiasis and other benign biliary diseases.

### Phase 3. Open Cholecystectomy—The Standard of Care

By the end of the 1920s, open cholecystectomy became the standard of care for nearly all elective inflammatory conditions of the gallbladder.^27–29^ It should be noted that the use of open tube cholecystostomy was taught as an alternative to cholecystectomy if the gallbladder dissection was dangerous or too tedious for the patient’s overall medical condition.^30^ This approach changed with the introduction of ultrasound-guided percutaneous drainage of the gallbladder many years later.^31^ Once the percutaneous technique of gallbladder drainage became more routine, patients who were medically unfit for cholecystectomy no longer required operative drainage of their gallbladder.^32^ As early as the 1930s, surgeons recognized the value of partial cholecystectomy as a bail-out option, further supplanting the need for operative tube cholecystostomy.^33,34^ As open cholecystectomy became the standard of care for most cases of cholecystitis or symptomatic cholelithiasis, debates have focused on perioperative care and intraoperative technique.

#### Perioperative Care

Timing of surgical intervention for acute cholecystitis was discussed early in the evolution of cholecystectomy^35^ with most surgeons believing that urgent operation was rarely required, and stabilization for several days was appropriate prior to operative intervention.^36–38^ Once antibiotics were established for the management of acute cholecystitis,^39^ some surgeons recommended that open cholecystectomy be delayed for at least 6 weeks to allow for improvement of both infection and inflammation prior to operative intervention.^40^ Operating between the 3^rd^ and 12^th^ day after symptoms of cholecystitis began was shown to be the least optimal timing for surgery.^41,42^ Ultimately it was demonstrated that early intervention (ie, within 48 hours of presentation) was safer than delayed intervention for most cases of cholecystitis.^43^

Once antibiotics were used for cholecystitis, there was little debate on their perioperative use.^44,45^ There was discussion on the type of antibiotic used, and this debate continued until randomized studies showed that cephalosporin-type antibiotics were equal or superior to others in most cases of cholecystitis.^46,47^

Postoperative care for cholecystectomy patients was designed as for a major laparotomy since these operations were commonly done through a large midline or right subcostal incision until the 1980s when mini-cholecystectomy was developed.^48^ From the 1920s until the 1950s, almost all open cholecystectomy patients had a nasogastric tube postoperatively and had a Foley catheter left in place for 24 hours after surgery. The nasogastric tube was removed after flatus or a bowel movement, and the initial diet was often clear liquids. The usual length of stay after open cholecystectomy was 7 to 14 days.^49–51^ Bed rest was commonly employed after all major laparotomies including open cholecystectomy, as there was concern that increased activity during the first 2 days after surgery would lead to failure of healing.^52^ During the late 1950s, some surgical investigators started to question the need for nasogastric suction on elective cholecystectomies, but the routine continued for most surgeons into the 1980s.^53,54^ By the 1980s, standard postoperative care following open cholecystectomy consisted of optional gastric decompression, early ambulation, and drain removal in 1 to 2 days.^55^

Despite improvement of mortality and morbidity of open cholecystectomy in the 1900s, alternatives, whether medical or surgical, continued to be investigated. In the 2^nd^ half of the 20^th^ century, oral administration of bile acids, such as chenodeoxycholic acid, was trialed to promote dissolution of gallstones as a non-surgical option for the management of gallstones.^56^ Recurrence following treatment as well as possible liver toxicity limited the use of chenodeoxycholic acid. Lithotripsy was also studied but fundamentally had the same issues, with high recurrence rates following therapy discontinuation.^57^ As such, benign biliary disease remained a surgical disease.

### Phase 4. Laparoscopic Cholecystectomy

The introduction of laparoscopic cholecystectomy was a watershed moment in the history of abdominal surgery.

Erich Muhe, a German surgeon, spurred by the advances of endoscopy and report of Kurt Semm laparoscopic appendectomy in 1980, performed the first reported laparoscopic cholecystectomy on September 12, 1985 with an endoscope (“Galloscope”).^58,59^ In 1987, Philippe Mouret performed the first video-assisted laparoscopic cholecystectomy^60^ and collectively with Francois Dubois and Jacque Perissat were responsible for what is called the French technique, where the patient is positioned in split-leg position and the surgeon stands between the patient’s legs.^61^ Shortly thereafter, laparoscopic cholecystectomy was performed in the United States^62^ and was rapidly adopted worldwide.

The rapid adoption of laparoscopic cholecystectomy has no precedent in surgical history. In the span of 5 years, laparoscopic cholecystectomy became the standard of care for benign biliary disease. Indeed, by 1992, 80% of all cholecystectomies in the United States were performed laparoscopically,^63^ and a 1993 National Institute of Health Conference statement referred to laparoscopic cholecystectomy as the treatment of choice for symptomatic gallstones.^64^

Patient demand and economic forces were important factors in the widespread and rapid adoption of laparoscopic cholecystectomy.^65,66^ Laparoscopic cholecystectomy was heavily publicized early on as a new technique for gallbladder removal with improved outcomes, leading to patients specifically requesting a laparoscopic approach.^67^ As cholecystectomy was the most common procedure done by general surgeons, surgeons were financially incentivized to learn the new technique or lose referrals.^68^

Interestingly, most surgeons cited perceived clinical advantages of laparoscopic cholecystectomy and the desire to keep up with the state-of-the-art as main reasons for early adoption,^63^ despite a lack of evidence of its superiority at that time. Indeed, the adoption of laparoscopic cholecystectomy was so swift that it became challenging to conduct randomized clinical trials due to difficulty in recruitment, patient drop-out after randomization, and perceived lack of equipoise.^69,70^ The first randomized clinical trial showing improved outcomes of laparoscopic cholecystectomy compared to conventional technique was published in 1992, after laparoscopic cholecystectomy was already widely adopted.^71^ Registry data, such as from the Southern Surgeon’s Club in 1991,^72^ provided early evidence that laparoscopic cholecystectomy was safe, in particular when performed by surgeons with experience in laparoscopy.

Early on, highly publicized reports on poor outcomes following laparoscopic cholecystectomy led to concern that some surgeons were poorly trained in the novel technique.^73^ The New York State Department of Health sent a memorandum to all New York state hospitals spelling out training requirements and new credentialing guidelines for laparoscopy. As a response, the Society of American Gastrointestinal and Endoscopic Surgeons was one of the first surgical bodies to offer training standards in laparoscopy and to publish guidelines for granting clinical privileges for laparoscopic surgery,^74^ paving the way for current training and credentialing standards.

Surgical dogma changed following the widespread adoption of laparoscopic cholecystectomy. Prior to 1990, general surgeons often taught their residents that incisions healed from “side-to-side, not end-to-end.” There are several meanings of this phraseology, but some suggested that incision size did not influence healing and recovery. In addition, it was generally believed that a larger incision resulted in a definitive diagnosis or a better technical outcome.^75^ After the introduction of laparoscopic cholecystectomy, it became clear that incision size was an important determinant of postoperative recovery.^71^ Despite longer operative times, laparoscopic cholecystectomy was associated with improved outcomes, shorter length of stay, and faster recovery compared with conventional or “mini” open cholecystectomy.^76–78^ In addition, the physiologic stress response of laparoscopic cholecystectomy is less than that of open surgery.^68,79,80^

The lessons learned from the adoption of laparoscopic cholecystectomy impacted all aspects of abdominal surgery. Before laparoscopy, postoperative nutrition was withheld until the return of bowel function, and nasogastric decompression was routine following abdominal surgery. Shorter stays after laparoscopic cholecystectomy challenged those paradigms, leading to earlier feeding of postsurgical patients and select use of nasogastric decompression in the ensuing years.^68^ Finally, the newly found expectation for a fast recovery after abdominal surgery led to shorter hospital stays following open cholecystectomy.^81^

Given the success of laparoscopic cholecystectomy, surgeons soon developed minimally invasive alternatives to other common open surgeries. Operations such as Nissen fundoplication,^82,83^ inguinal hernia,^84^ splenectomy,^85^ colectomy,^86^ and several other procedures became targets of minimally invasive surgery (MIS) innovation.^87^ The rest of abdominal surgery, including hepato-pancreatic-biliary surgery, urology, gynecology, and thoracic surgery, also made the move to smaller incisions.^88–91^ Within 10 years of the first laparoscopic cholecystectomy, nearly every surgical specialty was considering MIS approaches to disease. Today, 50% to 75% of elective abdominal and thoracic surgery are done with small incisions in high-income countries.^92^ MIS surgery is also expanding in middle- and low-income countries but at a slower rate.^93^

Management of the common bile duct and related pathologies continues to be debated. Following the transition to laparoscopic cholecystectomy, few surgeons would perform operative exploration to clear the common bile duct, in part owing to the complexity of performing laparoscopic common bile duct exploration and increased use of endoscopic retrograde cholangiopancreatography.^94^ However, over the last decade, laparoscopic common bile duct exploration has made a resurgence in adoption due to technical advances in cholangioscopy and stone extraction tools.^95^

Intraoperative cholangiography was introduced in the 1930s.^96^ There have been many retrospective studies debating the use of routine vs. selective cholangiography.^97–99^ This debate continues today as there is still a question if routine cholangiography is indicated to avoid retained common duct stones or if routine intraoperative imaging of the common duct can reduce the incidence of bile duct injury.^100,101^ In 2009, Ishizawa et al^102^ first described near-infrared fluorescent cholangiography using indocyanine green as an alternate means of visualizing biliary anatomy. Indocyanine green fluorescent cholangiography has since been increasingly used as an adjunct to white light alone, but no trials to date have compared whether near-infrared can prevent bile duct injury compared to intraoperative cholangiography or white light.^103,104^

Following the success of laparoscopic cholecystectomy, several improvements were brought forward with the hope of improving cosmesis, postsurgery quality of life, and shortening recovery.^105^ Single incision laparoscopic cholecystectomy, natural orifice transluminal endoscopic surgery cholecystectomy, and mini laparoscopic cholecystectomy were all pioneered in the late 1990s and early 2000s. However, none of these techniques has showcased a clear advantage over traditional laparoscopic cholecystectomy, limiting their success and adoption.

Robotic surgery was the next major evolution that occurred. The first robot-assisted laparoscopic cholecystectomy was performed in 1997.^106^ Since then, robot-assisted laparoscopic cholecystectomies have become widespread in the US, accounting for up to 25% of all cholecystectomies in select centers.^107^ Similar to the widespread adoption of laparoscopy for other abdominal surgeries, robotic surgery is now ubiquitous in every field of surgery.^108,109^ The use of a robotic platform to perform cholecystectomy remains debated with regards to safety^110,111^ and cost^112,113^ compared to laparoscopic cholecystectomy.

The introduction of laparoscopic cholecystectomy was a watershed moment in the history of surgery. The rapid adoption of laparoscopic cholecystectomy has no precedent in surgical history, and lessons learned from the adoption of laparoscopic cholecystectomy impacted all aspects of abdominal surgery. Laparoscopic cholecystectomy served as a gateway procedure for MIS innovations that have been occurring since the 1990s.

## Acknowledgments

I.J.A. and T.N.P.: Designed the study, reviewed the literature, and participated in the writing and revision of the manuscript.
